# Synaptic GABAergic transmission in the central amygdala (CeA) of rats depends on slice preparation and recording conditions

**DOI:** 10.14814/phy2.14245

**Published:** 2019-10-06

**Authors:** Elizabeth M. Avegno, Jason W. Middleton, Nicholas W. Gilpin

**Affiliations:** ^1^ Department of Physiology Louisiana State University Health Science Center New Orleans Louisiana; ^2^ Department of Cell Biology and Anatomy Louisiana State University Health Science Center New Orleans Louisiana; ^3^ Department of Neuroscience Center of Excellence Louisiana State University Health Science Center New Orleans Louisiana

**Keywords:** central amygdala, CRF, electrophysiology, neurotransmission, NMDG

## Abstract

The central nucleus of the amygdala (CeA) is a primarily GABAergic brain region implicated in stress and addictive disorders. Using *in vitro* slice electrophysiology, many studies measure GABAergic neurotransmission to evaluate the impact of experimental manipulations on inhibitory tone in the CeA, as a measure of alterations in CeA activity and function. In a recent study, we reported spontaneous inhibitory postsynaptic current (sIPSC) frequencies higher than those typically reported in CeA neurons in the literature, despite utilizing similar recording protocols and internal recording solutions. The purpose of this study was to systematically evaluate two common methods of slice preparation, an NMDG‐based aCSF perfusion method and an ice‐cold sucrose solution, as well as the use of an in‐line heater to control recording temperature, on measures of intrinsic excitability and spontaneous inhibitory neurotransmission in CeA neurons. We report that both slice preparation and recording conditions significantly impact spontaneous GABAergic transmission in CeA neurons, and that recording temperature, but not slicing solution, alters measures of intrinsic excitability in CeA neurons. Bath application of corticotropin‐releasing factor (CRF) increased sIPSC frequency under all conditions, but the magnitude of this effect was significantly different across recording conditions that elicited different baseline GABAergic transmission. Furthermore, CRF effects on synaptic transmission differed according to data reporting methods (i.e., raw vs. normalized data), which is important to consider in relation to baseline synaptic transmission values. These studies highlight the impact of experimental conditions and data reporting methods on neuronal excitability and synaptic transmission in the CeA.

## Introduction

The central nucleus of the amygdala (CeA) is a GABAergic brain region (Duvarci and Pare, 2015) with a demonstrated role in mediating physiological and behavioral responses to stress, fear, and/or drug‐related stimuli (Gilpin et al, 2015). CeA activity is modulated by excitatory and inhibitory input onto CeA neurons, and many studies have utilized *in vitro* electrophysiology to assess the impact on inhibitory transmission onto CeA neurons by various physiologically relevant stimuli, including stress exposure (e.g., Partridge et al, 2016), alcohol withdrawal (e.g., Roberto et al, 2004), and pain (e.g., Navratilova et al, 2019). In a recent study of alcohol dependence effects on periaqueductal gray (PAG)‐projecting CeA neurons (Avegno et al, 2018), we reported baseline spontaneous inhibitory postsynaptic current (sIPSC) frequency values that were much higher than those typically reported in the literature (e.g., Varodayan et al, 2016). To determine which experimental conditions may have contributed to these different results, we systematically compared two common methods of tissue preparation (using either NMDG‐based artificial cerebrospinal fluid [aCSF] or high‐sucrose aCSF solutions) and recording conditions (recording at room temperature, or maintaining temperature using an in‐line heater).

To promote the survival of neurons used in *in vitro* electrophysiological experiments, various protective cutting solutions are used during the brain slice preparation procedure. These are often aCSF recipes modified to replace sodium ions with an equimolar concentration of a substitute, such as sucrose. An ice‐cold high‐sucrose aCSF slicing solution (Aghajanian and Rasmussen, 1989) is commonly utilized for these studies, although alternate sodium substitutes are available. N‐methyl‐D‐glucamine (NMDG)‐based aCSF slicing solutions have been proposed as an alternative for tissue preparation, especially for older animals (Ting et al, 2014). Comparative experimental studies have demonstrated that tissue sectioning using room temperature NMDG aCSF solution preserves the viability of GABAergic interneurons in the cortex (Tanaka et al, 2008; Jiang et al, 2015), hippocampus, and thalamus (Pan et al, 2015). To our knowledge, no study has directly compared these two common slicing solutions on GABAergic transmission in the CeA. Given the extensive GABAergic interneuron connectivity in CeA (Duvarci and Pare, 2015), preservation of cell health is critical during electrophysiological recordings of GABAergic transmission in CeA.

A second common difference across slice electrophysiology experiments is the recording temperature, with studies performing experiments either at room temperature (e.g., Kirson et al, 2018; de Guglielmo et al, 2019), or using an in‐line heater to maintain recording aCSF at a more physiologically relevant temperature (e.g., Herman et al, 2013; Ji et al, 2017). Recording temperature affects the excitability of neurons *in vitro*, with greater activity in cells maintained near physiological temperatures (e.g., Cao and Oertel, 2005; Lee et al, 2005; Micheva and Smith, 2005; Graham et al, 2008; Baginskas et al, 2009), and was therefore included as an additional variable in our experiments. Similarly, recording temperature affects neurotransmitter release and subsequent alterations in current flow across the membrane, with increased temperature leading to increased neurotransmitter release and/or faster sIPSC/EPSC kinetics (Otis and Mody, 1992; Postlehwaite et al, 2007; Dixon et al, 2017). Together, our data demonstrate that slicing solution and recording temperature each altered (1) GABAergic transmission onto CeA neurons and (2) the effect of bath‐applied CRF on GABAergic transmission over time, which was further dependent on whether changes of sIPSC properties were reported as raw or normalized measures. Slicing solution had limited effects on intrinsic excitability of CeA neurons, although lower recording temperatures were associated with significantly decreased excitability of neurons. These results may be useful when planning *in vitro* electrophysiology experiments, and they highlight important considerations for experimental design and data presentation in these types of experiments.

## Materials and methods

### Animals

All procedures were approved by the Institutional Animal Care and Use Committee of the Louisiana State University Health Sciences Center and were in accordance with the National Institute of Health guidelines. Adult male Wistar rats (Charles River, Raleigh, NC, USA) were pair‐housed in a humidity‐ and temperature‐controlled (22°C) vivarium on a 12‐hr light/dark cycle (lights off at 8:00 am), with *ad libitum* access to food and water. Rats were acclimated for a minimum of 1 week before start of experiments. Average age of rats was 11 ± 0.25 weeks, with an average weight of 375 ± 10.9 g.

### NMDG brain slice preparation

Under isoflurane anesthesia, rats were transcardially perfused with room temperature (~22°C) NMDG artificial cerebrospinal fluid (aCSF) containing the following (in mM): 92 NMDG, 2.5 KCl, 1.25 NaH_2_PO_4_, 30 NaHCO_3_, 20 HEPES, 25 glucose, 2 thiourea, 0.5 CaCl_2_, 10 MgSO_4_·7 H_2_O, 5 Na‐ascorbate, 3 Na‐pyruvate (pH 7.3‐7.4, 300–305 mOsm). A total of 300‐µm‐thick coronal sections containing the CeA were collected in NMDG aCSF using a vibratome (Leica VT1200S, Nussloch, Germany). Sections were incubated in NMDG aCSF at 37°C for 12 min, then transferred to a room temperature holding aCSF solution containing the following (in mM): 92 NaCl, 2.5 KCl, 1.25 NaH_2_PO_4_, 30 NaHCO_3_, 20 HEPES, 25 glucose, 2 thiourea, 2 CaCl_2_, 2 MgSO_4_·7 H_2_O, 5 Na‐ascorbate, 3 Na‐pyruvate. Slices were allowed to recover for 1 hour prior to recording (Avegno et al, 2018).

### Sucrose brain slice preparation

Under isoflurane anesthesia, rats were decapitated, and brains were transferred immediately to an ice‐cold (~4°C) solution containing the following (in mM): 183 sucrose, 20 NaCl, 0.46 KCl, 1.34 NaH_2_PO_4_, 26 NaHCO_3_, 10 glucose, 1 MgCl_2_·6 H_2_O (pH 7.3‐7.4, 300–305 mOsm). A total of 300‐µm‐thick coronal sections containing the CeA were collected as described above and transferred to the holding aCSF solution. Sections were incubated in this solution at 37°C for 12 min, then transferred to room temperature. Slices were allowed to recover for 1 hour prior to recording.

### In vitro electrophysiology

Slices were visualized with oblique infrared light illumination**,** a w60 water immersion objective (LUMPLFLN60X/W, Olympus, Tokyo, Japan) and a CCD camera (Retiga 2000R, QImaging, Surrey, BC, Canada). Data were sampled at 10 kHz and Bessel filtered at 4 kHz using an acquisition control software package Ephus (Suter et al., 2010). CeA recordings were performed in the medial subdivision of the CeA (CeM). Sections were transferred to a recording aCSF solution containing the following (in mM): 130 NaCl, 3.5 KCl, 2 CaCl_2_, 1.25 NaH_2_PO_4_, 1.5 MgSO_4_·7 H_2_O, 24 NaHCO_3_, 10 glucose. Recording aCSF was maintained at 32–34°C using an in‐line heater (Warner Instruments, Hamden, CT; average temperature readout was 31.6 ± 0.87°C). A separate series of recordings were performed at room temperature (average temperature 22.1 ± 0.13°C).

Intrinsic excitability measures were recorded in current clamp mode using an internal recording solution containing the following (in mM): 140 K‐gluconate, 5 KCl, 0.2 EGTA, 10 HEPES, 2 MgCl_2_·6 H_2_O, 4 Mg‐ATP, 0.3 Na_2_‐GTP, 10 Na_2_‐phosphocreatine (pH 7.2‐7.3, 285–295 mOsm). Following break in and once a stable seal was achieved (usually ~ 3 min), resting membrane potential and spontaneous activity were recorded for 1 min. A series of current injections (0–140 pA, 20 pA increments, 1.5 sec duration) were delivered to determine rheobase values (defined as the minimum amount of current necessary to elicit an action potential), as well as the total number of action potentials generated in response to each current injection. Excitability was measured from each cell’s resting membrane potential. Input resistance was measured by injecting a 5 mV test pulse in voltage clamp, with neurons held at −60 mV. A minimum of three values were averaged for each neuron. Liquid junction potentials were not corrected during recordings. Experiments with a series resistance >30 MΏ or a >20% change in series resistance were excluded from analysis.

Spontaneous inhibitory postsynaptic current (sIPSC) recordings were performed using a KCl internal solution containing (in mM): 145 KCl, 5 EGTA, 5 MgCl_2_, 10 HEPES, 2 Na‐ATP, 0.2 Na‐GTP (pH 7.2‐7.3, 285‐295 mOsm), with cells clamped at −60 mV. To isolate sIPSCs, glutamate receptor antagonist kynurenic acid (2 mmol/L) and GABA_B_ receptor antagonist CGP 55845A (1 µmol/L) were included in the recording aCSF. Recordings were analyzed using Matlab R2019a (MathWorks, Natick, MA, USA). sIPSC recordings were analyzed in 1‐min bins. The final three bins were averaged to determine CRF‐induced change in sIPSC frequency and amplitude.

### Experimental design and statistical analysis

Statistical analysis was performed in Prism 7.03 (GraphPad Software, La Jolla, CA, USA) or in Matlab R2019a. Data were analyzed using two‐way ANOVA (Prism), or using three‐way ANOVA with repeated measures in one factor (Matlab). In the case of significant interaction and/or main effects, pairwise comparisons were probed with Tukey's post hoc analysis. A *P* value <0.05 was considered significant. All tests and variables are identified in the Results section.

## Results

### Intrinsic excitability

**Figure 1:**
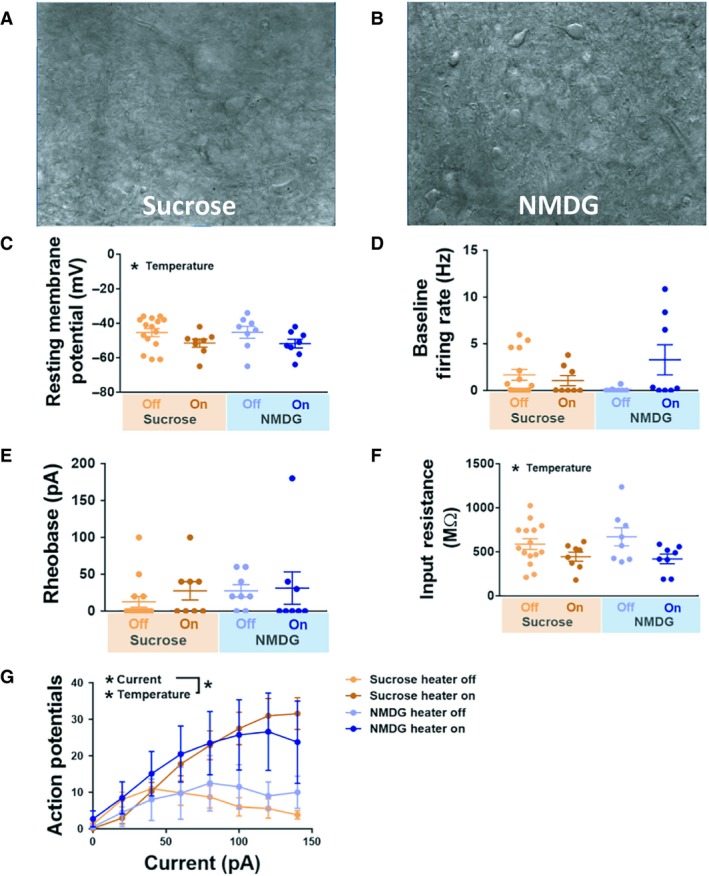
Effects of slice preparation and recording temperature on intrinsic excitability measures on CeA neurons. (A) Representative brightfield image of CeA‐containing tissue section from sucrose preparation, demonstrating reduced visibility of neurons relative to slices derived from NMDG preparation (B). (C), Resting membrane potential of CeA neurons from slices prepared in NMDG (blue) and sucrose (orange), recorded with (dark) and without (light) the heater on. *Indicates main effect of recording temperature; *P* < 0.05, two‐way ANOVA. (D) Baseline firing rat of each neuron recorded. (E) Rheobase values for each neuron recorded. (F) Input resistance values for each neuron recorded. *Indicates main effect of recording temperature; *P* < 0.05, two‐way ANOVA. (G) Number of action potentials generated in response to increasing current injections for neurons under each condition. *Indicates main effect of current and recording temperature, as well as an interaction between the two; *P* < 0.05, three‐way ANOVA. Values in (C‐G) shown as mean ± SEM. The number of cells and rats for each condition were as follows: NMDG‐heater on, eight cells from three rats; NMDG‐heater off, eight cells from four rats; sucrose‐heater on, eight cells from four rats; sucrose‐heater off, 15 cells from 5 rats.

We first evaluated the effects of slicing solution and recording temperature on intrinsic excitability of CeA neurons. Compared to slices prepared using sucrose solution (Fig. 1A), we observed enhanced visibility of neurons prepared using NMDG solution (Fig. 1B). The number of cells and rats for each condition were as follows: NMDG‐heater on, eight cells from three rats; NMDG‐heater off, eight cells from four rats; sucrose‐heater on, eight cells from four rats; sucrose‐heater off, 15 cells from 5 rats. In both slicing solution groups, resting membrane potential (RMP) was significantly affected by recording temperature, with more depolarized potentials in room temperature neurons compared to “heater on” cells (Fig. 1C; Table 1). A two‐way ANOVA revealed a significant effect of temperature on RMP (*F*(1,35) = 5.02; *P* = 0.032), but not slicing solution (*F*(1,35) = 0.00032; *P* = 0.99), with no significant interaction (*F*(1,35) = 0.0051; *p* = 0.94). Baseline firing rates were not affected by temperature (*F*(1,35) = 2.24; *P* = 0.14), or slicing solution (*F*(1,35) = 0.14; *P* = 0.71; Fig. 1D). A significant interaction (*F*(1,35) = 4.93, *P* = 0.033) was found, although Tukey’s multiple comparisons test revealed no significant pairwise difference between any of the conditions (*P *> 0.05 in all cases). Similar results were found when performing analysis on active neurons (baseline firing rates greater than 0 Hz) only. Among spontaneously active neurons, baseline firing rate was not affected by temperature (*F*(1,18) = 2.43; *P* = 0.14), or slicing solution (*F*(1,18) = 0.18; *P* = 0.68), with no significant interaction (*F*(1,18) = 2.78; *P* = 0.11; two‐way ANOVA). Rheobase values were not affected by temperature (*F*(1,35) = 0.54; *P* = 0.47), or slicing solution (*F*(1,35) = 0.54; *P* = 0.47), with no significant interaction (*F*(1,35) = 0.19, *P* = 0.66; Fig. 1E). These effects remained when performing analysis on inactive neurons (rheobase values greater than 0 pA) only. Among inactive neurons, rheobase was not affected by temperature (*F*(1,13) = 1.66; *P* = 0.22), or slicing solution (*F*(1,13) = 0.17; *P* = 0.68), with no significant interaction (*F*(1,13) = 0.87; *P* = 0.37; two‐way ANOVA). In both slicing solution groups, input resistance was significantly affected by recording temperature, with higher input resistance values in room temperature neurons compared to “heater on” cells (Fig. 1F; Table 1). A two‐way ANOVA revealed a significant effect of recording temperature (*F*(1,35) = 7.38; *P* = 0.010), with no effect of slicing solution (*F*(1,35) = 0.16; *P* = 0.69), and no interaction (*F*(1,35) = 0.56; *P* = 0.46). Capacitance values were not significantly affected by recording temperature (*F*(1,36) = 1.71; *P* = 0.20) or slicing solution (*F*(1,36) = 0.77; *P* = 0.39), with no significant interaction (*F*(1,36) = 0.032; *P* = 0.86; Table 1). In response to a series of increasing current steps, recording temperature had a significant effect on action potential firing, with greater excitability in “heater on” relative to “heater off” cells (*F*(1,25) = 4.33; *P* = 0.048; between‐subjects comparison, three‐way ANOVA; Fig. 1G), with no significant effect of slicing solution (*F*(1,25) = 0.15; *P* = 0.70; between‐subjects comparison, three‐way ANOVA). Within‐subjects analysis revealed a significant effect of current (*F*(7,175) = 27.26; *P* < 0.0001), with a significant temperature X current interaction (*F*(7,175) = 10.40; *P* < 0.0001), as well as a significant three‐way interaction (*F*(7,175) = 2.62; *P* = 0.014). These data indicate that recording temperature, but not slicing solution, affects the intrinsic excitability of CeA neurons.

**Table 1:** Resting membrane potential, input resistance, and capacitance values across recording conditions. Data shown as mean ± SEM.

Recording Condition	Resting Membrane Potential (mV)	Input Resistance (MΩ)	Capacitance (pF)
Sucrose‐heater off	−45.4 ± 2.4	588.7 ± 60.2	45.4 ± 5.2
Sucrose‐heater on	−51.5 ± 2.4	445.9 ± 51.0	55.5 ± 10.2
NMDG‐heater off	−45.3 ± 3.5	671.7 ± 102.6	36.0 ± 7.4
NMDG‐heater on	−51.8 ± 2.5	420.9 ± 54.7	56.3 ± 13.7

### Spontaneous inhibitory postsynaptic currents (sIPSCs)

**Figure 2:**
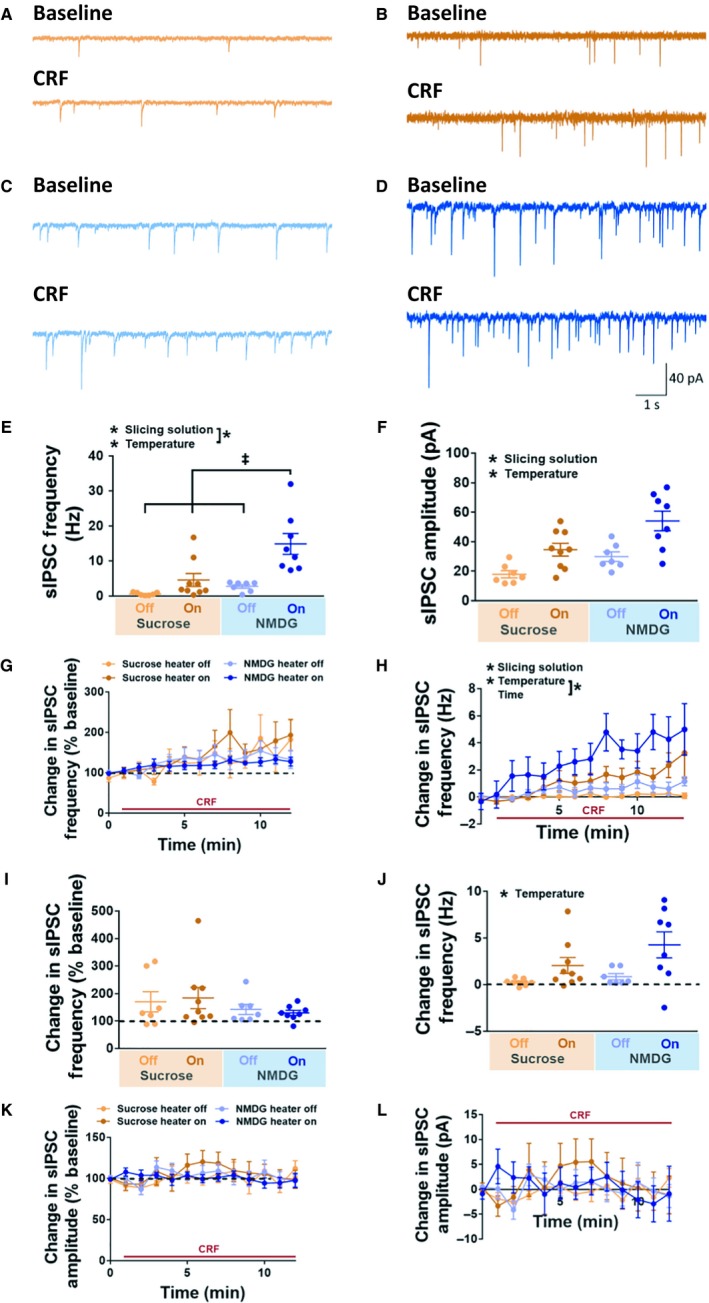
Effects of slice preparation and recording temperature on spontaneous inhibitory postsynaptic currents (sIPSCs) recorded in CeA neurons. Representative sIPSC traces at baseline (top) and in the presence of CRF (bottom) from CeA neurons in the sucrose‐heater off (A), sucrose‐heater on (B), NMDG‐heater off (C), and NMDG‐heater on (D) conditions. Scale bar shown in (D) describes panels A‐D. (E) Baseline sIPSC frequency of CeA neurons recorded under each condition. *Indicates main effect of slicing solution and recording temperature; *P* < 0.05, two‐way ANOVA; ^‡^ *P* < 0.05 relative to NMDG‐heater on, Tukey’s multiple comparisons test. (F) Baseline sIPSC amplitude of CeA neurons recorded under each condition. *Indicates main effect of slicing solution and recording temperature; *P* < 0.05, two‐way ANOVA. (G) Change in sIPSC frequency in the presence of CRF, expressed as a percentage of baseline. No significant effect of time, slicing solution, or recording temperature indicated by three‐way ANOVA. (H) Change in sIPSC frequency in the presence of CRF, expressed as a raw change from baseline. *Indicates main effect of slicing solution, recording temperature, and time, as well as an interaction between the time and recording temperature; *P* < 0.05, three‐way ANOVA. (I) Average CRF‐induced change in sIPSC frequency, expressed as a percentage of baseline. (J) Average CRF‐induced change in sIPSC frequency, expressed as a raw change from baseline. *Indicates main effect of recording temperature; *p* < 0.05, two‐way ANOVA. Relatively small changes in sIPSC frequency in (J) appear as large changes in (I) for those neurons with a low baseline sIPSC frequency, and vice versa. (K) Change in sIPSC amplitude in the presence of CRF, expressed as a percentage of baseline. No significant effect of time, slicing solution, or recording temperature indicated by three‐way ANOVA. (L) Change in sIPSC amplitude in the presence of CRF, expressed as a raw change from baseline. No significant effect of time, slicing solution, or recording temperature indicated by three‐way ANOVA. Data in (E‐L) shown as mean ± SEM. The number of cells and rats for each condition were as follows: NMDG‐heater on, eight cells from six rats; NMDG‐heater off, seven cells from four rats; sucrose‐heater on, nine cells from eight rats; sucrose‐heater off, seven cells from six rats.

We next evaluated the effects of slicing solution and recording temperature on spontaneous GABAergic transmission onto CeA neurons. The number of cells and rats for each condition were as follows: NMDG‐heater on, eight cells from six rats; NMDG‐heater off, seven cells from four rats; sucrose‐heater on, nine cells from eight rats; sucrose‐heater off, seven cells from six rats. Spontaneous inhibitory postsynaptic currents (sIPSCs) were recorded in the presence of glutamate receptor blocker kynurenic acid and GABA_B_ blocker CGP 55845A. We observed a significant effect of both slicing solution (higher frequency in NMDG vs. sucrose conditions; *F*(1,27) = 10.49; *P* = 0.0032) and recording temperature (higher frequency in heater on vs. off condition; *F*(1,27) = 17.58; *P* = 0.0003) on baseline sIPSC frequency of CeA neurons, with a significant interaction (*F*(1,27) = 4.49; *P* = 0.043; two‐way ANOVA, Fig. 2A–E). Tukey’s multiple comparisons test revealed a significant difference between NMDG‐heater on condition and each other recording condition (NMDG‐heater off, sucrose‐heater on, and sucrose‐heater off; *P* < 0.05 in all cases). Baseline sIPSC amplitude was significantly affected by both slicing solution (higher amplitude in NMDG vs. sucrose conditions; *F*(1,27) = 11.33; *p* = 0.0023) and recording temperature (higher amplitude in heater on vs. off conditions; *F*(1,27) = 19.2; *P* = 0.0002), with no significant interaction (*F*(1,27) = 0.65; *P* = 0.42; two‐way ANOVA, Fig. 2F).

In the presence of CRF (200 nmol/L), we observed an increase in sIPSC frequency over time, when data were expressed as raw change in sIPSC frequency from baseline (Fig. 2H), but not when expressed as a percentage of baseline (Fig. 2G). A three‐way ANOVA for CRF effects on raw sIPSC frequency revealed a significant effect of slicing solution (*F*(1,26)  = 6.63; *p* = 0.016; between‐subjects comparison) and recording temperature (*F*(1,26) = 13.08; *P* = 0.0013; between‐subjects comparison; Fig. 2H). The three‐way ANOVA for CRF effects on raw sIPSC frequency also revealed a significant effect of time (*F*(12,312)  = 12.87; *P* < 0.0001; within‐subjects comparison), and a significant temperature x time interaction (*F*(12,312) = 2.21; *P* = 0.011; Fig. 2H). A separate three‐way ANOVA revealed no significant effect of any variable on percent change from baseline (Fig. 2G). Average CRF‐induced change in sIPSC frequency, expressed as percentage of baseline, did not significantly differ among groups (Fig. 2I; recording temperature: *F*(1,27) = 0.00013, *P* = 0.99; slicing solution: *F*(1,27) = 1.91, *P* = 0.18; interaction: *F*(1,27) = 0.21, *P* = 0.63; two‐way ANOVA). Recording temperature had a significant effect on average CRF‐induced change in sIPSC frequency, expressed as a raw change from baseline, with a larger change in “heater on” relative to “heater off” cells (*F*(1,27 = 8.36; *P* = 0.0075; two‐way ANOVA), but there was no effect of slicing solution (*F*(1,27) = 2.40, *P* = 0.13) or interaction effect (*F*(1,27) = 0.37, *P* = 0.37; Fig. 2J). We observed that, for those neurons with the smallest baseline sIPSC frequency values, relatively modest raw increases in sIPSC frequency (Fig. 2J) translated to rather large percentage increases in frequency (Fig. 2I), and vice versa. There were no significant effects of time, slicing solution, or recording temperature on sIPSC amplitude, whether data were expressed as a percentage of baseline (Fig. 2K) or as a raw change from baseline (Fig. 2L).

## Discussion

To our knowledge, these experiments are the first to systematically compare the effect of slicing solution and recording temperature on inhibitory neurotransmission and intrinsic excitability of CeA neurons. We find that recording temperature, but not slicing solution, impacts the excitability of CeA neurons, with resting membrane potential, input resistance, and current‐induced action potentials significantly impacted by recording temperature. These findings agree with published reports of temperature effects on the excitability of neurons in the dorsal horn of the spinal cord (Graham et al., 2008), cortex (Lee et al., 2005), and cerebellum (Baginskas et al, 2009). In an effort to minimize differing variables across slice preparation conditions, we limited sucrose incubation time to 12 min, to match that of the NMDG preparation methods, but it should be noted that studies utilizing a sucrose slicing solution typically incubate sections for a longer duration (generally ≥30 min). Another important variable affecting spontaneous activity of neurons is extracellular K^+^ concentration; here, we used a K^+^ concentration commonly used in slice electrophysiology studies (e.g., Varodayan et al., 2016).

Both slicing solution and temperature affected spontaneous GABAergic transmission in the CeA, with significant effects on baseline sIPSC frequency and amplitude. Together, these two variables likely account for the discrepancy between our previously published baseline sIPSC frequency values (Avegno et al., 2018) and those typically reported in the literature (e.g., Varodayan et al, 2016). In the presence of CRF, we observed an increase in sIPSC frequency in neurons from each recording condition, with no significant effect on sIPSC amplitude, similar to what has been previously reported (Roberto et al, 2010). We report here that CRF induced the largest raw changes in sIPSC frequency in neurons with the highest baseline sIPSC frequency values, but these changes appeared much smaller when data were expressed as a percentage of baseline. We also found that CRF‐induced small raw changes in sIPSC frequency in neurons with low baseline sIPSC frequency values, but these changes appeared much larger when data were expressed as a percentage of baseline. These findings highlight the impact of data reporting methods on the apparent effect of bath‐applied drugs. Changes in sIPSC frequency or amplitude are often expressed as a normalized percentage of baseline to account for variability in baseline values, but raw changes in sIPSC frequency are relevant because this measure represents the actual amount of current passing through the cell membrane. Reporting sIPSC changes only in normalized values would potentially obscure the fact that important drug‐mediated changes in synaptic transmission critically depend on brain slice preparation solution. Furthermore, reporting both measures (raw changes and normalized effects) is good practice for integrating new results with prior literature.

Higher inhibitory neurotransmission observed in NMDG‐prepared slices may be reflective of a greater preservation of cell health, rather than a general increase in cellular activity, as (1) the intrinsic excitability of recorded neurons was not significantly impacted by slicing solution and (2) other reports suggest NMDG‐based aCSF solutions preserve the survival of GABAergic neurons in the cortex (Tanaka et al, 2008; Jiang et al, 2015), hippocampus, and thalamus (Pan et al, 2015). This preservation of neuronal health has been attributed to sodium ion replacement to reduce excitotoxic swelling, as well as mitigation of oxidative stress by inclusion of cellular antioxidants in the NMDG aCSF (Ting et al, 2014). Notably, components included to reduce edema and oxidative damage, including HEPES and thiourea (Brahma et al, 2000; MacGregor et al, 2001), are absent from the high‐sucrose cutting aCSF. Our results support that in CeA slices, preservation of neuron health is ideal for the characterization of synaptic or circuit properties, such as spontaneous inhibitory transmission. Despite the apparent differences in global slice health, individual neurons viable for recordings exhibit no apparent differences in intrinsic excitability regardless of slicing solution (i.e., sucrose vs. NMDG). Collectively, these experiments suggest that, for *in vitro* electrophysiological experiments aimed at recording GABAergic transmission in the CeA, using an NMDG‐based aCSF solution for slice preparation and using an in‐line heater to maintain slice temperature may lead to optimal conditions for recording.

## Conflict of interest

NWG owns shares in Glauser Life Sciences, Inc., a start‐up company with interest in development of therapeutics for treatment of mental illness. All other authors declare no competing financial interests.
